# Mesenchymal stem cell-derived exosome: a promising alternative in the therapy of Alzheimer’s disease

**DOI:** 10.1186/s13195-020-00670-x

**Published:** 2020-09-14

**Authors:** Mengtian Guo, Zhenyu Yin, Fanglian Chen, Ping Lei

**Affiliations:** 1 Department of Geriatrics, Tianjin Medical University General Hospital, Tianjin, China; 2 Tianjin Geriatrics Institute, Tianjin Medical University General Hospital, Tianjin, China; 3 Tianjin Neurological Institute, Tianjin, China

**Keywords:** Mesenchymal stem cell, Alzheimer’s disease, Exosome immunomodulation, Therapeutics

## Abstract

Alzheimer’s disease (AD) has been a devastating public health with the development of global aging. Approaches for reducing the current AD epidemic are becoming a primary focus of human healthcare due to the lack of achieved lasting and complete remission strategies to treat AD with the characteristics of heterogeneity and complexity. Exosomes, which is the new emerging approach to intercellular communication, provide novel perspective on identified therapeutic strategies of AD. Mesenchymal stem cell-derived exosomes (MSC-exos) are emerging to be an appealing therapeutic tool for AD, with the donor-derived properties and the characteristics of minimal immunogenicity, effortless storage, nature delivery vehicles, and low risks of tumor formation based on the previous researches. In this review, we elaborate the mechanism of MSC-exos in the treatment of AD and discuss limitations in the clinical application.

## Introduction

Alzheimer’s disease (AD) is the most common cause of dementia in the elderly, which contributes to 60–80% of total dementia population [1]. What is worse, the incidence of AD is rising continually [2]. As a neurodegenerative disease, AD is associated with high disability rate, resulting in more severe cognitive impairment than aging, which brings heavy burden to public administration and caregivers [1, 3].

AD is characterized by increased deposition of β-amyloid peptides and aggregation of hyperphosphorylated tau in neurofibrillary tangles (NFT) [4]. However, clinical symptoms vary with the region of brain injury. Typical clinical symptoms include progressive decline of episodic memory and executive functions [5]. In contrast, atypical clinical symptoms general occur in non-memory domains, presenting agnosia, aphasia and disturbed executive function [6]. From the therapeutic level, the existing medicine approved by Food and Drug Administration (FDA) for AD patients include three cholinesterase inhibitors (e.g., ChEIs; donepezil, rivastigmine, and galantamine) and an uncompetitive NMDA receptor 2 modulator (memantine). Unfortunately, the aforementioned medicine merely aims to improve quality of life and extend lifespan, but fail to halt disease progression [7]. Seeking novel therapeutic strategies for AD is urgent.

In recent years, mesenchymal stem cells (MSCs) have attracted much attention as the potential cell-based therapeutic tools due to its ability of migrating and mediating damage repair. MSCs facilitate neurological recovery and neo-angiogenesis through the secretion of neurotrophins and angiogenesis regulatory factors [8–10]. The ability of immunomodulatory effect, migratory capability, and regenerative potential of MSCs are confirmed in multiple disease models, such as atopic dermatitis, myocardial infarction, traumatic brain injury, and diabetes nephropathy [11–14]. However, accumulating researches suggest that the biodistribution of MSCs in the target organs is rare, and its therapeutic effect appears to be a consequence of the paracrine action [15–17]. The particles in the secreted proteins of MSCs are identified as exosomes through electron microscopy and proteomic analysis [18].

Exosomes (also called small extracellular vesicles (EVs)) are considered as a subtype of extracellular microvesicles with a diameter of 30–100 nm, including lipid bilayer membrane structure, and released by variety of cell types [19, 20]. Investigators primarily proposed the presence of extracellular vesicles in mammalian tissues or body fluids as early as in 1960s [21, 22], and the exosomes were considered as the unnecessary proteins for a long time [23, 24]. In fact, exosomes are able to reflect the state of the parent cell [25] and mediate intercellular communication through transporting biologically active cargo (including proteins, lipids, and nucleic acids) to recipient cells both in physiological and pathological conditions [26, 27]. Given the powerful biological functions, exosomes have been studied for applications as vaccines, immunosuppressant, or stimulators of repair and differentiation process [28].

Herein we outline the role of exosomes in AD treatment. Additionally, we elaborate the therapeutic properties of MSC-exos in AD and discuss the advantages and challenges of MSC-exos as a novel cell-free therapeutic agent.

## Exosome biogenesis, secretion, and uptake

**Fig. 1:**
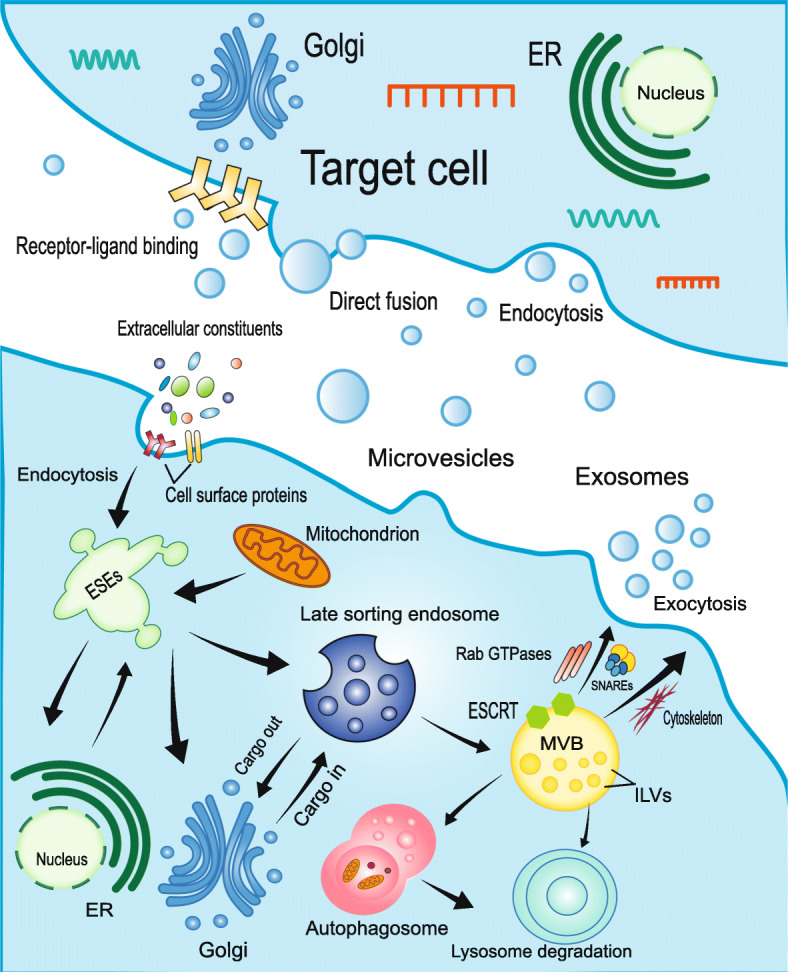
The mechanism of exosome biogenesis, secretion, and uptake. Microvesicles are released through plasma membrane budding. Extracellular constituents and cell surface proteins form the early sorting endosome (ESEs) through endocytosis along with plasma membrane budding inward. The endoplasmic reticulum (ER), Golgi, and mitochondria are involved in the maturation of ESEs through fusion. ESEs give rise to late sorting endosome and multivesicular bodies (MVBs) in succession. Partial MVBs release their vesicles into the extracellular space as exosomes. Others are transported to lysosomes for degradation through fusing with autophagosomes or not. The formation of ILVs is mainly controlled by endosomal sorting complex required for transport (ESCRT). Rab GTPases, SNAREs, and cytoskeleton are involved in the regulation process of exosome secretion. Exosomes can deliver cargo to recipient cells by three methods: endocytosis, direct membrane fusion, and receptor-ligand binding

Enormous efforts were invested to clarify the mechanism of exosome biogenesis, secretion, and uptake (Fig. 1). Originally, extracellular constituents and cell surface proteins form the early sorting endosome (ESEs) through endocytosis along with plasma membrane budding inward. During the maturation process of ESEs, intraluminal vesicles (ILVs) begin to compose through invagination of the limiting endosomal membrane [29]. The formation of ILVs is controlled by several molecular machineries, mainly regulated by the machinery complex termed endosomal sorting complex required for transport (ESCRT) [30]. The ESCRT mechanism is composed of approximately 30 proteins which assemble into four complexes (ESCRT-0, ESCRT-I, ESCRT-II, ESCRT-III) and associated proteins (e.g.,Vps4, Alix, Tsg101) involved in the formation of ILVs [31, 32]. ESCRT-0 sequesters ubiquitinated cargo proteins, ESCRT-I/II/III induce membrane deformation, and Vps4 complex ensure vesicle scission and recycling of the ESCRT-III complex [33]. Another pathway of exosomes biogenesis is generated independently of ESCRT machinery mechanisms, involving tetraspanins, ceramides, heat-shock proteins (HSPs), cholesterol, and phosphatidic acids [19]. Lipid-mediated RNA loading into exosomes depends on self-organizing lipid and cargo domains [34]. Subsequently, cytoplasmic molecules such as proteins, lipids, and RNAs are encapsulated into the lumen and accumulated within the late endosome, thus forming multivesicular bodies (MVBs) [35]. The endoplasmic reticulum and Golgi complex are involved in the process. Partial MVBs fuse with the plasma membrane through cytoskeletal and microtubule network of the cell, ultimately releasing their vesicles into the extracellular space as exosomes, while others are transported to lysosomes for degradation through fusing with autophagosomes or not [36]. Compared with the degradative MVBs, the secreted MVBs contain more of ceramides [37, 38]. It has been reported that the different fates of MVBs may be related to the simultaneous existence of subpopulations in cells [19]. Since the endosome pathway is involved in exosome formation and release, proteins such as tetraspanins (CD9, CD63, and CD81), flotillin, Alix, and TSG101 are used as exosomal markers [19]. Additionally, exosomes are characterized by their high concentration of lipid raft components, such as ceramide and sphingomyelin [39, 40].

Although the process of exosome release remains elusive, it is widely accepted that Rab GTPases, soluble N-ethylmaleimide-sensitive factor attachment protein receptors (SNAREs), and cytoskeleton are involved in the regulation process [41]. Rab GTPase proteins are involved in transferring vesicles between intracellular compartments and regulation of MVB fusion with plasma membrane for exosome release [42, 43]. For example, Rab27 is able to alter MVB morphology and dock to the plasma membrane. Rab35 is located on the surface of oligodendroglia cells and regulate the docking of endocytic vesicles and plasma membrane [42, 44]. The cytoskeleton exhibit significant polarity distribution inside the cells, allowing variation in the distribution of MVBs. Targeting actin polymerization may indirectly promote exosome secretion [24]. Soluble N-ethylmaleimide-sensitive factor attachment protein receptors (SNAREs) are considered as central catalysts of intracellular membrane fusion [45]. SNARE complex facilitates fusion of two opposing membranes in a zipper-like manner [46].

It has been reported that endocytosis is the primary pathway for exosome uptake. In addition, exosomes can deliver cargo to recipient cells through direct membrane fusion and receptor-ligand binding [47]. The steps of exosome uptake mainly include targeting, entry, and delivery of content [24]. In particular, exosome uptake may be affected by crucial factors such as temperature and size distribution. Low temperature induces proteolytic cleavage of exosomal proteins thereby inhibiting the release of exosomes [48]. Smaller exosomes are more easily taken up by cells. Moreover, tetraspanin membrane proteins and intercellular adhesion molecule (ICAM-1) are considered to promote the uptake of exosomes. Of note, the currently known biogenesis pathways are not specific for exosomes and not suitable for all cell types yet [24].

## Role of exosomes in AD

To date, the exact etiology of AD is not particularly clear. Amyloid cascade hypothesis plays dominant role in explaining the genesis and progression of AD [49]. However, accumulating evidence demonstrate that amyloid cascade hypothesis fail to explain the pathophysiological mechanism of AD completely, and tau hypothesis, mitochondrial cascade hypothesis, and neuroinflammation hypothesis are proposed successively [50, 51]. It is worth noting that exosomes are involved in the process of AD [52].

Firstly, brain-derived exosomes in peripheral blood have shown great potential to be an ideal “liquid biopsy” for AD (Table 1). Exosomes derived from blood are characterized by low invasive diagnostic procedures with high sensitivity and specificity [61]. Clinical diagnosis of AD depends on symptoms, neuropsychological testing, lumbar puncture, and neuroimaging [62, 63]. Intriguingly, brain-derived exosomes can penetrate the blood-brain barrier into the peripheral blood circulation, but the concentration is lower than that of cerebrospinal fluid. To overcome these limitations, investigators enrich brain-derived exosomes from plasma through immunoprecipitation methods. In addition, several studies suggest that due to the overlapping levels of Aβ1–42, T-tau, and p-tau181 in CSF, it cannot effectively distinguish AD patients from other types of dementia patients [64]. Intriguingly, a multiple center study confirms the correlation between the levels of AD-associated protein in CSF and blood [55]. In the previous researches, lysosomal and synaptic proteins levels of neuron-derived exosomes (NDEs) are useful for the preclinical risk prediction of conversion from mild cognitive impairment (MCI) to dementia [56, 57]. The reductions in NDE levels of functionally specialized synaptic proteins may reflect the severity of AD progression [58]. Furthermore, the levels of complement proteins in exosomes derived from astrocytes (ADEs) are apparently associated with stage of the disease [59]. Cargo proteins in plasma ADEs are significantly higher than those in NDEs, which may be a potential target for BACE-1 inhibitors [56]. Moreover, large cohort studies are needed to assess the diagnosis utility of exosomes, and standardized process for preparation and biomarker quantification is still greatly challenging [65].

**Table 1:** Exosomes as AD biomarkers in discussed studies

Source	Body fluid	Isolation methods	Validation techniques	Direction of protein change	Ref.
Neuronal	Plasma	EXOQ + anti-L1CAM immunocapture	TEM, NTA	**ADC and AD**: P-T181-tau, P-S396-tau, and Aβ1–42 ↑, NRGN, REST ↓ compared to CNC and stable MCI patients	[53]
Neurally	Plasma or serum	EXOQ + anti-NCAM immunocapture	NTA	**AD**: total Tau, P-T181-tau, P-S396-tau and Aβ1–42 ↑compared to controls **FTD**: P-T181-tau and Aβ1–42 ↑ compared to controls	[54]
Neuronal	Plasma or CSF	EXOQ + anti-NCAM immunocapture	TEM, WB	**AD**: Aβ42, T-tau, and P-T181-tau ↑ compared to aMCI and control groups The level of each exosomal biomarker was highly correlated with that in CSF	[55]
Neuronal	Plasma	EXOQ + anti-L1CAM immunocapture	NTA, TEM, WB	**AD and FTD**: synaptophysin, synaptopodin, synaptotagmin-2, and neurogranin ↓ compared to controls **AD**: GAP43, synapsin 1 ↓ synaptotagmin, synaptophysin, and neurogranin were correlated with MMSE or ADAS-cog	[56]
Neurally	Plasma	EXOQ + anti-L1CAM immunocapture	NTA	**AD**: cathepsin D, LAMP-1, ubiquitinylated proteins ↑, and HSP70 ↓ compared to controls and FTD	[57]
Neuronal	Plasma	EXOQ + anti-L1CAM immunocapture	NTA, TEM, WB	**AD**:NPTX2, NRXN2α, AMPA4, NLGN1 ↓ **Preclinical period**: NRXN2α, AMPA4, and NLGN1 ↓ compared to controls	[58]
Astrocyte	Plasma	EXOQ + anti-ACSA-1 immunocapture	NTA, TEM, WB	**AD**: complement proteins, IL-6, TNF-α, IL-1β ↑; complement regulatory proteins (CD59, CD46, DAF), complement receptor type 1 ↓ compared to controls	[59]
Astrocyte	Plasma	EXOQ + anti-ACSA-1 immunocapture	NTA	**AD**: BACE-1, (s)APPβ ↑, GDNF ↓ compared to controls **FTD**: compared to controls n. d.	[60]

↑ higher; ↓ lower; *n. d.* no difference compared to control, *EXOQ* ExoQuick exosome precipitation solution, *L1CAM* neural adhesion protein, *TEM* transmission electron microscope, *NTA* Nanoparticle Tracking Analysis, *MCI* mild cognitive impairment, *ADC* MCI converting to AD, *REST* repressor element 1-silencing transcription factor, *NRGN* neurogranin, *ADAS-cog* AD assessment scale-cognitive subscale, *CNC* cognitively normal controls, *FTD* frontotemporal dementia, *GAP43*growth-associated protein 43, *MMSE* Mini-Mental State Examination, *LAMP-1* lysosome-associated membrane protein 1, *HSP70* heat-shock protein 70, *ACSA-1* antihuman glutamine aspartate transporter, *NPTX2* neuronal pentraxin 2, *NRXN2α* neurexin 2α, *AMPA4* GluA4-containing glutamate, *NLGN1* receptor and neuroligin 1, *TNF-α* tumor necrosis factor-α, *DAF* decay-accelerating factor, *BACE-1* β-site amyloid precursor protein-cleaving enzyme 1, *(s)APP* soluble amyloid precursor protein, *GDNF* glial-derived neurotrophic factor

Secondly, exosomes are considered as a potential vehicle for drug delivery in AD. As a natural biological agent, exosomes possess homing capabilities to transport active molecules between cells with favorable biocompatibility, and transiently modulate functions of targeted cells [66]. It has reported that MSC-exos can interact with target cells through different mechanisms. For example, MSC-exos can directly bind to membrane receptors to internalize their contents in target cells. Moreover, MSC-exos are able to deliver biologically active substances into target cells by fusion with the plasma membrane. What is more, exosomes are able to cross the blood-brain barrier (BBB) easily to enhance intracranial drug concentration [67]. Compared with conventional approaches of administration, exosome delivery avoids some complications, including intracranial infection, nonspecific absorption, and drug toxicity [68]. Lipid bimolecular structure of exosomes contributes to improving transport efficiency and supporting the load of hydrophobic or hydrophilic drugs [69]. Moreover, exosomes are characterized by low immunogenicity and long circulating half-life, which can prevent “therapeutic cargo” from rapid degradation [68]. Previous studies indicate that exosomes can deliver drugs or siRNA to the brain of AD mice [68, 70]. However, there are evidences which demonstrate that predominant localization of intravenously administered exosomes is in the spleen and liver, with lower signals in the brain [71]. In order to increase the concentration of intravenously administered exosomes, the surface of exosomes can be modified by connecting peptides. For example, exosomes from engineered dendritic cells expressing membrane protein Lamp2b can bind to neuron-specific Rabies virus glycoprotein (RVG) peptide. These results showed that the cognitive function of AD transgenic mice injected with engineered MSC-exos was significantly improved [72].

Additionally, exosomes are involved in the process of pathogenic protein clearance. Beta-amyloid peptide and tau, two symbolic pathological proteins in AD, have been suggested to be associated with the neuronal damage and death, leading to gradual decline in memory and cognitive impairment [73]. Previous studies suggest that exosomes can reduce the deposition of Aβ in different ways. For example, NDEs facilitate the conversion of Aβ to nontoxic amyloid fibrils via driving conformational changes [74]. Inhibiting neutral sphingomyelinase 2 (nSMase2) contribute to reducing the conversion of sphingomyelin to ceramide and ultimately reduce the deposition of amyloid plaque [75]. Glycosphingolipids (GSLs) on the surface of exosomes can bind to Aβ, accelerating the clearance of amyloid depositions and reducing synaptic toxicity [76]. Notably, the evidence demonstrate that exosomes are not only involved in accelerating clearance of aggregated proteins, but also contribute to propagation of pathogenic proteins [77]. The propagation of pathogenic protein in AD is along neuroanatomically connected areas of the brain, which is considered to occur in a prion-like manner [78]. However, the transmission mechanism of the pathogenic protein is still controversial and further research is needed.

Finally, exosomes play a role in promoting neurogenesis and reducing cognitive impairment. However, the specific cellular and molecular mechanisms of these processes are still unclear. It has been reported that NDEs are able to regulate the number of AMPA receptors for glutamate transmission or induce synaptic pruning by the overexpression complement component 3 of microglia cells, indicating that NDEs are involved in the process of synaptic plasticity. Additionally, neuronal EVs are involved in the regulation of miRNA content and neuronal excitability [79, 80]. Several studies have reported the existence of exosomes of non-neuronal cell origin. In the state of oxygen and glucose deprivation, oligodendrocyte-derived EVs contribute to elevate neuronal viability via uptake by neurons. Additionally, oligodendrocyte-derived exosomes are involved in the regulation of oxidative stress and increment of firing rate of neurons [81]. Exosomes derived from microglial are confirmed to regulate synaptic activity and transmit neuroprotective substances between cells [82]. Microvesicles derived from microglial are involved in the process of upregulating synaptic activity by promoting ceramide and sphingosine production [83]. Moreover, the surface of ADEs which expressed a novel glycoprotein (Synapsin-1) facilitates neurite outgrowth in the state of oxidative stress [84], which is a benefit for the recovery of nerve impairment. Interestingly, it was reported that exosomes can restore nerve function by increasing nerve density and inhibiting oxidative stress damage, rather than generating new neurons [85].

## MSC-exos isolation, storage, and administration

MSCs are a class of adult multipotent stromal cells with self-renewal capabilities. They can be isolated from a variety of sources, including bone marrow (BM-MSCs), adipose tissue (AD-MSCs), umbilical cord (UC-MSCs), amniotic fluid, placenta, and peripheral blood [86–91]. The International Society for Cellar Therapy defines that MSCs should express the cell surface markers CD73,CD90, and CD105, but not hematopoietic and endothelial antigens (CD14 or CD11b, CD19 or CD79α, CD34, CD45, HLA-DR) [92]. As stem cells, MSCs contribute to differentiating into cell lineages of mesodermal origin (e.g., osteoblasts, chondrocytes, and adipocytes) through stimulation in vitro [93]. However, this issue is still controversial. Some studies indicate that MSCs are able to differentiate into other cell types, such as endothelial cells, neural cells, glial cells, cardiomyocytes, and hepatocytes [94–96]. Differences of cell sources, isolation protocols, and culture environment can affect the properties of MSCs (Table 2). In the previous research, autologous MSCs are different from those of healthy donors, which will influence the outcomes of treatment [97]. Compared with other cell types, MSCs are able to produce higher doses of exosomes. The production of exosomes is related to the proliferation rate of MSCs. The cell density and content of secreted growth factors in MSCs cultured with three-dimensional (3D) spheroid were more than those with traditional monolayer culture [98]. Unlike cell transplantation, exosomes do not need to be cultured to a suitable density before transplantation, which lays the foundation for the treatment of acute diseases [97].

**Table 2:** Overview the characteristics of the described exosome isolation methods

Methods	Working principle	Advantages	Disadvantages	Ref.
**UC**	Differential centrifugationbased on density	Reduced protein contamination High purity	Low yield, difficult to separate particles of similar size, expensive equipment support	[99–101]
**Density gradient**	Based on density additional steps after centrifugation	High purity	Low yield, time-consuming	[102]
**SEC**	Based on hydrodynamic radius	Good reproducibility, rapid and mild Reduced protein contamination	Low sample recovery	[103, 104]
**Filtration**	Based on molecular mass and size	Simple and time-saving	Potential to alter structural integrity, low sample recovery	[105, 106]
**Immunoaffinity**	Antibody capture	High selectivity and purity, no need for additional equipment support	High cost, nonspecific binding	[107]
**Commercial kits**	Precipitation with chemicals	High yield	High protein contamination	[53, 58]
**AF4**	Laminar flow	Classification of EV subtypes, efficiently	Low sample recovery and repeatability	[108]
**Nano-FCM**	High-resolution flow cytometry	High-fidelity sorting	Simultaneous detection of multiple EVs, expensive equipment support	[109]
**Microfluidics**	Based on physical or mechanical characteristics	Low sample volumes, rapid and high purity	Not suitable for large sample processing, expensive equipment support	[110]

*UC* ultracentrifugation, *SEC* size-exclusion chromatography, *AF4* asymmetric flow field-flow fractionation, *FCM* flow cytometry

Given the heterogeneity and complexity of exosomes, different isolation methods have been discovered (Table 3). (1) Ultracentrifugation (UC) is to obtain purified exosomes by repeated differential centrifugation, filtration, and washing, which was considered as the most widely used method in the current researches. However, membrane damage of exosomes may occur during centrifugation. Additionally, UC requires long time and expensive equipment support [120]. For further exosome purification, an additional density gradient step can be added on the basis of UC. However, the drawbacks of this method are low yields [121]. (2) Size-exclusion chromatography (SEC) is used to separate exosomes based on hydrodynamic volume. This process is filtered through a stationary-phase column of porous beads with approximately the same size as the particle of interest [122]. The characteristic of SEC is time-saving, low cost, and good repeatability, but the recovery rate and purity of exosomes are reduced. (3) Filtration is a separation method based on the molecular mass and size of particles. After the initial filtration, additional ultrafiltration and repeated washing processes are needed to remove the impurities. The advantage of filtration is it is simple, gentle, and time-saving. However, exosomes may cause morphological changes due to squeezing when passing through the filter membrane, resulting in a lower recovery rate [123]. (4) Immunoaffinity isolation methods are based on the specific antigen–antibody interactions to capture exosomes, such as coated magnetic bead immunoaffinity pull-down or filter paper chromatography [124]. The advantage of this method is specific purification. However, the antibodies may have weaknesses, such as short lifespan, nonspecific binding, and cross-reactivity. (5) Commercial kits are popular in the current research, including ExoQuick™, Exo-Flow™, and Total Exosome Isolation Precipitation. The yield of exosomes isolated with commercial kits is substantial, but the protocols are complicated and the purity of exosomes is low [124, 125]. (6) Nano-flow cytometry (Nano-FCM) has been proved to be efficient in the isolation and quantification of exosomes [126]. Compared with traditional flow cytometry, Nano-FCM could detect the particles below the size of 200 nm. The disadvantages of FCM are the multiple nano-particles detected at the same time, resulting in high signal and inaccurate measurement [127, 128]. In addition, several emerging technologies are gradually being applied, such as microfluidic EV isolation techniques and asymmetric flow field-flow fractionation (AF4) [106]. Taken together, isolation methods with few separation steps will result in high yield but low purity of exosomes. To date, the standards for exosome isolation have not been unified. Therefore, we can combine multiple methods to improve the separation efficiency [129].

**Table 3:** Application of MSC-derived EVs in Alzheimer’s disease

Source	Extraction method	Administration scheme	Results	Ref.
hucMSC	ExoQuick	Male 7 months old AβPP/PS1 mice 30 μg/100 μl, i.v., every 2 weeks, four times	Alleviate neuroinflammation and Aβ deposition	[111]
ADSCs	Ultracentrifuge	Co-culture N2a cells with ADSCs in serum-free medium for 2–3 days	Carry active NEP Decrease Aβ levels	[112]
RVG-BM-MSC	Ultracentrifuge	7-month-old APP/PS1 mice; B6C3-Tg 5 × 10^11^ /100 μl, i.v. monthly for 4 months	Improve learning and memory capabilities Reduce plaque deposition Normalize inflammatory cytokine levels	[72]
BM-MSC	Ultracentrifuge	APP/PS1 mice 100 μg/5 μl, i.c.v., once per 2 days for 2 weeks	Alleviate iNOS expression Improve cognitive behavior Reduce synaptic impairment and LTP	[113]
PC-BM-MSC	ExoQuick	7-month-old APP/PS1 mice 150 μg/80 μl, i.v., biweekly for 4 months	Improve learning and memory capabilities Restore synaptic dysfunction Regulate inflammatory responses	[114]
hMSC	Ultracentrifuge	hippocampal cells incubated with HMSC-EVs (6 × 10^8^ particles) for 22 h, add AβOs (500 nM) for 2 h	Rescue oxidative stress Block synapse damage Carry active catalase	[115]
BM-MSC	Ultracentrifuge	Co-culture MSC-exo with hippocampal neurons in serum-free medium for 24 h, add AβOs (500 nM) for 6 h/24 h	Protect neurons against AβO-induced oxidative stress and synapse damage	[116]
ADSCs	Ultracentrifuge + ExoQuick	Incubate NSCs from TG2576 mice with ADSC-exo (200 μg/mL) for 24/48 h	Reduce Aβ levels and neuronal apoptosis	[117]
BM-MSC	Ultracentrifuge	5-month-old APP/PS1 mice 22.4 μg/4 μL, i.c.v.	Reduce Aβ burden and the amount of dystrophic neurites Carry neprilysin	[118]
hUMSCs	Ultracentrifuge	Nine-month-old male APP/PS1 mice 2 mg/ml, i.c.v., continuously at 0.25 μL/h for 14 days	Reduce Aβ generation, inflammation and oxidative stress Inhibit microglia activity Improve spatial learning and memory function	[119]

*hucMSC* human umbilical cord mesenchymal stem cells, *ADSCs* human adipose tissue-derived mesenchymal stem cells, *CM* conditioned medium, *hMSC* human Wharton’s jelly mesenchymal stem cells, *BM-MSC* bone marrow-derived mesenchymal stem cells, *RVG* rabies viral glycoprotein, *LTP* long-term potentiation, *i.v.* intravenous injection, *i.c.v.* intraventricular injection, *PC* hypoxia-preconditioned, *AβOs* amyloid beta oligomers, *NSCs* neuronal stem cells

Several studies indicate that the storage temperature and freeze-thaw times are able to affect the therapeutic utility of exosomes. After comparing, researchers suggest that storage temperature at − 80 °C is the most suitable condition for long-term preservation of exosomes [130]. Repeated freezing and thawing may not only cause inaccurate assessment of the size and quantity of exosomes, but also induce the loss of cargo. Therefore, the minimized freeze-thaw cycles will be beneficial to maintain the characteristics and functions of exosomes [131].

To date, the administration route and dosage of MSC-exos are still inconclusive. Experimental results indicate that the route or schedule of administration may significantly influence the dosage of therapeutic MSC-exos [132]. In addition, further research is also needed to determine the frequency of administration based on the duration of exosomes in vivo. Of note, it has been shown that repetitive or sustained delivery of MSC-exos significantly enhances their biovailability and efficacy [133]. Importantly, the optimal therapeutic scheme should be comprehensively determined according to the clinical condition [132].

## Therapeutic properties of MSC-exos in AD

**Fig. 2:**
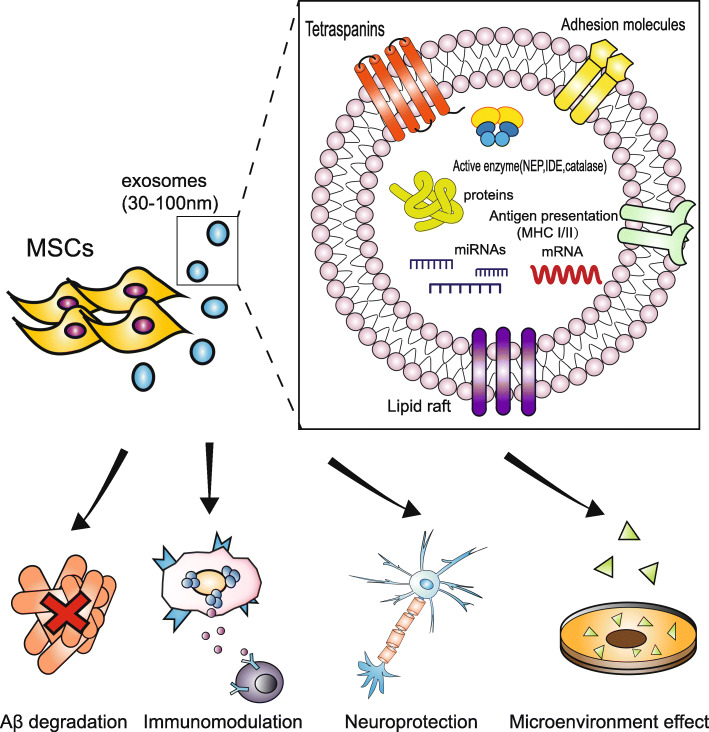
The cargo and therapeutic role of MSC-exos in AD. MSC-exos are a subtype of extracellular microvesicles characterized by a lipid bilayer membrane structure with a diameter of 30–100 nm. Exosomes generally include active cargos such as proteins, lipids, and nucleic acids. In particular, MSC-exos carry Aβ degradation-related enzyme (NEP, IDE). The arrows show the therapeutic role of MSC-exos in AD, including Aβ degradation, immunomodulation, neuroprotection, and microenvironment effect

MSC-derived microvesicles were initially proposed to promote cell proliferation and enhance anti-apoptotic ability of tubular epithelial cells in acute tubular injury model in 2009 [134]. Accumulating evidence demonstrate that MSC-exos possess the ability of modulating immunity, promoting Aβ degradation, and ameliorating neurological impairments (Fig. 2). In this context, MSC-exos are considered to be a potential option for the treatment of AD (Table 2).

### MSC-exos promote Aβ degradation

In the previous studies, β-amyloid is considered as the production of proteolytic cleavage of the amyloid precursor protein (APP) by β- and γ-secretases [135]. Aβ monomers are relatively nontoxic, while oligomers are the reverse. Actually, the production and degradation of Aβ is balanced in the normal brain, while the abnormal accumulation results in metabolic imbalance. Once the clearance capacity of lysosomes or glial cells is overloaded, the pathogenic protein will be released into extracellular space and propagate across different brain areas through the exosome pathway. Clearance of pathogenic proteins has been shown to be beneficial in the treatment of AD [136]. Neprilysin (NEP) and insulin-degrading enzyme (IDE), zinc metallopeptidase, are related to Aβ degradation in the brain [137]. In 2000, researchers injected radiolabeled synthetic Aβ 1–42 peptide into rat hippocampus and observed that the brain-derived NEP was capable of proteolyzing peptide subsequently [138]. In NEP- or IDE-deficient mice, endogenous Aβ levels were elevated in a gene dose-dependent manner [139, 140]. In the recent research, MSC-exos with NEP and IEP activity reduced the deposition of Aβ plaques of AD transgenic mice through intravenous injection [111, 112]. Therefore, MSC-exos play an important role in the degradation of Aβ, reflecting potential of MSC-exos in the treatment of AD.

### Immunomodulatory effects of MSC-exos

It is widely indicated that the pathogenesis of AD is closely related to the immune system. To our knowledge, classical neuroimmune cell-to-cell communication is interacted with membrane [79]. MSCs play an immunomodulatory role through low levels of class II major histocompatibility complex (MHC-II) and co-stimulatory molecules on the cell surface [141]. Of note, MSC-exos containing the immunologically active molecules can regulate the immune cells. For example, MSC-exos contribute to inhibiting the proliferation and differentiation of lymphocytes [142]. Moreover, MSC-exos are involved in inducing lymphocytes to differentiate into an anti-inflammatory type. To our knowledge, MSC-exos are able to induce conversion of T helper type 1 (Th1) cells into T helper type 2 (Th2) cells, reduce potential of T cells to differentiate into interleukin 17-producing effector T cells (Th17), and elevate the expression of regulatory cells (Tregs) [143, 144]. Additionally, several studies have demonstrated that inflammatory cytokines and proteins contained in MSC-exos have immunomodulatory effects.

Neuroinflammation is emerging as a central pathological process in AD [145]. Excessive accumulation of Aβ in the brain triggered the neuroinflammation process. MSC-exos contribute to immune regulation and neuroinflammation amelioration in pathological abnormal areas, as well as significantly improve the spatial learning ability and memory impairments in AD transgenic mice [72, 144]. Moreover, MSC-exos contribute to inducing anti-inflammatory effects by the inhibition of activated microglia, reactive astrocytes, and the release of cytokine [146].

In addition, MSC-exos are able to suppress inflammatory response through regulating enzyme activity. Aβ induce nitric oxide synthase (NOS) in glial cells and then release high levels of nitric oxide (NO). NO induces neurotoxicity via inhibition of mitochondrial respiration, resulting in neuronal cell death [147]. In this context, MSC-derived extracellular vesicles (EVs) reduce the expression of iNOS in vitro and alleviate the deficits of APP/PS1 mice in long-term potentiation (LTP) to CA1 synaptic [113].

Several previous studies suggested that MSC-exos affect post-transcriptional gene expression and ensue protein expression in the target cells via the delivery of miRNAs. miRNAs, small noncoding RNAs, play a key role in regulating several biological processes such as growth, inflammation, and angiogenesis. Exosomes containing miRNAs released by MSCs can inhibit the activity of immune cells and enable their phenotypic conversion into anti-inflammatory. For example, microglia plays the role of first innate immune defensive line in brain. When activated, microglia exhibit two different polarized phenotypes namely M1/M2 [97, 148]. In vivo and vitro models, elevating the levels of miR-124-3p in microglial exosomes, result in the inhibition of neuronal inflammation by promoting anti-inflammatory M2 polarization and contribute to alleviating neurodegeneration [98, 105]. In a previous study, dysfunctional miRNAs are related to AD via the observation of altered miRNA expression profiles in AD brains [149]. The levels of miRNA-21 are significantly reduced in the status of chronic inflammation and apoptosis. However, MSC-exos contains high levels of miRNA-21, which contributes to reducing inflammation and apoptosis [150]. Furthermore, exosomes from preconditioned MSCs not only effectively increased the level of miR-21, but also inhibited NF-κB activation and STAT3 expression in APP/PS1 mice. Overexpression of miR-21 contributes to rescue memory deficits and regulate pathologic process [114]. In addition, it has been confirmed that MSC-exos containing miR-142-3p, miR-223-3p, and miR-126-3p regulate dendritic cell maturation and promote their anti-inflammatory potential in other disease models. MSC-exos can inhibit the expression of TRAF6 and IRAK1 via delivery of miRNA-146a to macrophages, resulting in the downregulated phosphorylation of NF-κB and reduction of inflammatory factors. Moreover, it is well known that some miRNAs contained in MSC-EVs are obviously related to their therapeutic properties. For example, miR148a, miR532-5p, and miR378 participate in angiogenesis, cellular transport, proteolysis, and apoptosis. miR-21, miR-17-92, and miR-133b are linked to neural damage, and miR-145 is related to the processes of cellular differentiation [151].

### Neuroprotective effects of MSC-exos

Synapse dysfunction, another pathologic hallmark, generally appears in the early stage of AD, which is directly related to cognitive impairment. As suggested by recent findings, hMSC-EVs protect hippocampal neurons via blocking oxidative stress and synapse damage exposed to amyloid beta oligomers (AβOs) [115]. Additionally, researchers found that the mechanisms of neuroprotection by MSC-derived EVs are related to containing the endogenous active antioxidant enzyme, catalase [116]. The expression levels of synaptic proteins are capable of reflecting the function of synapses to some extent. Synapsin 1 and PSD95 are synaptic protein involved in nerve signal transmission and maintaining synaptic integrity. The exosomes derived from hypoxia-preconditioned MSCs significantly enhance the expression of synaptic proteins (Synapsin 1 and PSD95) [114]. Neurite growth and synaptogenesis are controlled in terms of neuronal development. ADSC-derived exosomes decrease the levels of apoptotic proteins (such as p53, Bax, pro-caspase-3, and cleaved-caspase-3) and simultaneously downregulate the expression of anti-apoptotic proteins in vivo and vitro AD models. Moreover, ADSC-derived exosomes were found to increase the neurite growth of neuronal stem cells (NSCs) from the transgenic mice TG2576 by measuring the length and number of neurites [117]. In addition, MSC-exos are capable of transferring miR-133b into astrocytes and neurons and promote the recovery of neural function [152]. Taken together, MSC-exos play a potential role in promoting neurite outgrowth and suggest the possibility of clinical treatment in AD [118].

### MSC-exos are affected by extracellular environment

Given different cell sources, the characters of exosomes vary according to content of cargos, which are affected by the physiological situation and extracellular environment [119]. Hence, it is essential to consider the efficacy of MSC-exos with environment changes. To our knowledge, MSCs are capable of strong plasticity as stem cells. Several recent studies have assessed that pretreated MSCs in vitro affect the contents and biological activities of secreted exosomes. It was reported that exosomes obtained from hypoxia-preconditioned MSCs (PC-MSCs) are able to enhance the therapeutic effect in AD transgenic mice [114]. The advantages of pretreatment group are mainly reflected in improving learning and memory capabilities, alleviating Aβ accumulation, increasing synaptic protein expression, and suppressing inflammatory response. In recent research, MSCs were pretreated with GW627368X (a prostaglandin E2 receptor 4 antagonist), which induced MSC-EVs containing anti-inflammatory cytokines and neuron-supporting proteins. The induced MSC-EVs suppress astrogliosis and microglia infiltration, restore BBB integrity, and elevate memory and learning ability in the hippocampus damage model [153]. Intriguingly, the abovementioned therapeutic effects failed to appear in MSC-EVs under conventional culture condition. Additionally, it is worth noting that MSCs likely modify the characteristics of exosomes in pathological conditions. In turn, there are studies which show that MSC-EVs contributed to altering cellular metabolic microenvironment through carrying biologically active components [154]. Therefore, the continuous optimization of MSC pretreatment methods, at least to some extent, may enhance the therapeutic potential of MSC-exos in AD.

## Clinical trials of MSC-exos in AD

Despite the results of previous experiment are promising, few on-going or completed clinical studies have explored the potential role of MSC-exos in clinical trials [96]. After consulting the database, there is a clinical trial now listed at www.clinicaltrials.gov utilizing MSC-exos. Researchers from the Shanghai Jiao Tong University School of Medicine decide to evaluate the safety and effectiveness of MSC-exos in patients with mild to moderate dementia (NCT04388982). This clinical trial plans to recruit 9 patients to participate in the study. Patients will be given three doses of low, medium, and high (5 μg, 10 μg, 20 μg) MSC-exos twice a week, respectively, for 12 weeks via nasal drip as planned. In the primary stage of trails, patients will be measured for the functions of liver or kidney and treatment-related adverse events. Cognitive function, quality of life, and neuroimaging will be evaluated during the secondary stage of trails. Although the clinical trials of MSC-exos in the treatment of AD have not yet been done, promising results have been confirmed in other diseases (NCT03562715, NCT04356300, and NCT04134676). It is encouraging that MSC-exos can ameliorate inflammation and improve kidney function of grade III-IV chronic kidney disease (CKD) patients in a phase II/III clinical pilot study. Of note, during the 1 year follow-up, no adverse events related to the administration of MSC-exos were found in subjects [155]. In addition, the therapeutic effects of MSC-exos after stroke have been explored in the clinical trials conducted at Isfahan University of Medical Sciences (NCT03384433). Subjects were given allogenic MSC-exos transfected by miR-124 via a stereotactic technique. During the 1-year follow-up, subjects have been monitored for treatment-related adverse events, such as brain edema, deteriorating stroke, stroke recurrences, and hemorrhagic transformation. This study was completed in December 2019 and the results are unannounced. Therefore, based on the experience of successful clinical trials in other diseases, the results of MSC-exos in AD clinical trials are promising.

## Advantages and challenges for MSC-exos in the application of AD therapy

Accumulating evidence suggest that MSC-exos possess superior safety profile, anti-inflammatory effects, minimal immunogenicity, and low risks of tumor formation [99, 156, 157]. Unlike MSCs, exosomes cannot replicate, which contribute to avoiding uncontrolled division. This feature of exosomes greatly reduces the risk of tumor formation during the process of proliferation [151]. Exosomes can also avoid mutations and DNA damage caused by cell transplantation [158]. Due to nanometric size, MSC-exos reduce the possibility of vascular obstruction and cross blood-brain barrier easier [159]. Moreover, the surface of MSC-exos could be modified and exploited into engineered exosomes, which can bind the ligands with target specific cells and escape immune responses [129]. The low immunogenicity of MSC-exos makes allogeneic applications possible [160]. In addition, engineered exosomes can increase the drug concentration of target organs and achieve personalized treatment effects. From the production perspective, mesenchymal stem cells (MSCs) possess biological characteristics of multi-differentiation potential and are effortless to proliferate and store. In addition, MSCs can not only produce high quantities of exosomes, but also ensure that the composition does not change significantly, which are appropriate to large-scale production [161].

As a therapeutic agent, MSC-exos still face great challenges. The main reasons may be attributed to the following points. (1) The isolation, storage, and purification protocols of MSC-exos still need continuous majorization and standardization to enhance the comparability and reproducibility. Since the conventional isolated methods mainly depend on density and size, some substances (lipoproteins, virus, etc.) may overlap with their characteristics, resulting in incomplete removal. Clinical applications depend on time-saving, low-cost, and convenient methods, but the existing separation methods obviously do not meet these conditions. In addition, in order to promote the development of effective biomarkers for exosomes, sensitive, accurate, and rapid quantitative methods are essential. (2) The therapeutic effects of MSC-exos in promoting neurite growth may vary depending on the source of MSCs [162]. However, due to the significant differences in the route of administration, dosage, separation protocols, and disease model, it is difficult to determine the specific source of MSC-exos with higher therapeutic potential. (3) Some detrimental cytokines of MSCs are secreted through paracrine effects, suggesting that it is essential to clarify the contents of exosomes and eliminate interference from unknown secretory factors. (4) Several critical technological issues have not yet been resolved, such as side effect of drugs, optimal dosage, and route of administration [163]. Due to the complex biological composition of exosomes, the difference between exosomes and single medication should be considered in the application process [160]. More experimental works are needed to be carried out before extensive clinical trials. Determining the specific therapeutic molecules of MSC-exos is worth noting in further research [132]. (5) The value of exosome as delivery vehicles required to be fully evaluated and directly compared with existing viral vectors and biosynthetic vectors [160].

## Conclusion

Although much effort has been invested in AD, the achievements seem to be unsatisfactory [164]. The previous works in AD therapeutics focused on the amyloid hypothesis. Unfortunately, almost all phase III clinical trials ended in failure, which suggest that other existing important pathological mechanisms are involved in the cause of AD [165]. As a novel cell-free therapeutic agent, MSC-exos have unparalleled advantages over cell-based therapy, which are considered to be a promising alternative in the therapy of AD.

## Data Availability

Data sharing is not applicable to this article as no datasets were generated or analyzed during the current study.

## Abbreviations

AD: Alzheimer’s disease
MSC-exos: Mesenchymal stem cell-derived exosomes
FDA: Food and Drug Administration
ChEIs: Cholinesterase inhibitors
BM: Bone marrow
AD-MSCs: Adipose tissue-derived mesenchymal stem cells
UC-MSCs: Umbilical cord mesenchymal stem cells
BDNF: Brain-derived neurotrophic factor
VEGF: Vascular endothelial growth factor
NGF: Nerve growth factor
EVs: Extracellular vesicles
ESEs: Early sorting endosome
ILVs: Intraluminal vesicles
ESCRT: Endosomal sorting complex required for transport
HSPs: Heat shock proteins
MCI: Mild cognitive impairment
MVBs: Multivesicular bodies
Aβ: β-amyloid
ADEs: Exosomes derived from astrocytes
NFT: Neurofibrillary tangles
NDEs: Neuron-derived exosomes
BBB: Blood-brain barrier
RVG: Rabies virus glycoprotein
SNAREs: Soluble N-ethylmaleimide-sensitive factor attachment protein receptors
GSLs: Glycosphingolipids
nSMase2: Neutral sphingomyelinase 2
MAPS: Misfolding-associated protein secretion
NEP: Neprilysin
IDE: Insulin-degrading enzyme
ADSCs: Human adipose tissue-derived mesenchymal stem cells
MHC-II: Class II major histocompatibility complex
Th1: T helper type 1
Th2: T helper type 2
Th17: Interleukin 17-producing effector T cells
PBMCs: Peripheral blood mononuclear cells
iNOS: Inducible nitric oxide synthase
UC: Ultracentrifugation
NOS: Nitric oxide synthase
FCM: Flow cytometry
LTP: Long-term potentiation
SEC: Size-exclusion chromatography
AF4: Asymmetric flow field-flow fractionation
APP: Amyloid precursor protein
PC: Preconditioned
CKD: Chronic kidney disease
eGFR: Estimated glomerular filtration rate
UACR: Urinary albumin creatinine
